# Keeping abreast with long non-coding RNAs in mammary gland development and breast cancer

**DOI:** 10.3389/fgene.2014.00379

**Published:** 2014-10-31

**Authors:** Herah Hansji, Euphemia Y. Leung, Bruce C. Baguley, Graeme J. Finlay, Marjan E. Askarian-Amiri

**Affiliations:** ^1^Auckland Cancer Society Research Centre, University of AucklandAuckland, New Zealand; ^2^Department of Molecular Medicine and Pathology, University of AucklandAuckland, New Zealand

**Keywords:** long non-coding RNA, breast cancer, mammary gland development, gene regulation, epigenetics

## Abstract

The majority of the human genome is transcribed, even though only 2% of transcripts encode proteins. Non-coding transcripts were originally dismissed as evolutionary junk or transcriptional noise, but with the development of whole genome technologies, these non-coding RNAs (ncRNAs) are emerging as molecules with vital roles in regulating gene expression. While shorter ncRNAs have been extensively studied, the functional roles of long ncRNAs (lncRNAs) are still being elucidated. Studies over the last decade show that lncRNAs are emerging as new players in a number of diseases including cancer. Potential roles in both oncogenic and tumor suppressive pathways in cancer have been elucidated, but the biological functions of the majority of lncRNAs remain to be identified. Accumulated data are identifying the molecular mechanisms by which lncRNA mediates both structural and functional roles. LncRNA can regulate gene expression at both transcriptional and post-transcriptional levels, including splicing and regulating mRNA processing, transport, and translation. Much current research is aimed at elucidating the function of lncRNAs in breast cancer and mammary gland development, and at identifying the cellular processes influenced by lncRNAs. In this paper we review current knowledge of lncRNAs contributing to these processes and present lncRNA as a new paradigm in breast cancer development.

## INTRODUCTION

Ribonucleic acids were long thought to act mainly as carriers of genetic information between DNA and the protein-synthesizing machinery of the cell. Whole genome technology, including deep sequencing and microarray analyses, has demonstrated that coding regions constitute only 2% of the human genome. On the other hand, 70–90% of the genome is transcribed (Bertone et al., 2004; Carninci et al., 2005; Birney et al., 2007; Kapranov et al., 2010; Djebali et al., 2012). Initially regarded as ‘junk’, these transcripts have been found to possess many regulatory roles and represent a largely unexplored area of cell biology.

Non-coding RNAs (ncRNAs) are defined as those RNAs which are transcribed from the genome but do not encode proteins. They are divided into two broad classes based on their function: housekeeping and regulatory. Housekeeping ncRNAs include ribosomal, transfer, small nuclear, and small nucleolar RNAs (snoRNAs). These ncRNAs are constitutively expressed and crucial to the operation of vital cellular functions. Regulatory ncRNAs are differentially expressed according to tissue and stage of development or disease process. These regulatory ncRNAs are divided into two broad classes based on their size. The first class of regulatory ncRNAs comprises short transcripts <200 nt long and includes microRNA (miRNA), small interfering RNA (siRNA), piwi interacting RNA (piRNA), transcription initiation RNA (tiRNA), and small cajal body-specific RNA (scaRNA). They control a number of developmental and physiological pathways and have been shown to be deregulated in disease processes (Mattick and Makunin, 2006; Carthew and Sontheimer, 2009). The second class of regulatory ncRNAs is long ncRNAs (lncRNA), which is defined as those transcripts >200 nt. Despite making up the majority of the human transcriptome, they were initially dismissed as products of leaky transcription or evolutionary noise due to their lack of open reading frames and poor sequence conservation. However, lncRNAs have now been found to display key characteristics that confirm their roles as functional molecules, and a significant number of papers has been published in recent years focusing on their function in normal development and disease. They are mainly transcribed by RNA polymerase II and the genomic loci of many of them contain chromatin marks, consistent with those of transcribed genes, at their promoters and gene bodies such as H3K9ac, H3K4me3, and H3K36me3. They are also frequently polyadenylated and undergo splicing of exons, demonstrating that they require post-transcriptional processing to form mature transcripts. Furthermore, lncRNA expression is regulated by well characterized transcription factors (reviewed in Guttman and Rinn, 2012).

In general, lncRNAs show poor sequence conservation, due to their rapid evolution, but display conserved tissue specificity and function (Necsulea et al., 2014). Recent work has provided information on the origin and evolution of lncRNAs, suggesting that lncRNA emerge *de novo* from transposable elements or non-exonic sequences (Derrien et al., 2012; Kapusta et al., 2013). Sequences conserved between different species might be expected to show conserved functions, but a recent study has shown significant differences in stem cell-specific transcription factor binding sites between mouse and human (Odom et al., 2007). This observation suggests that mouse models cannot always be used for studying human lncRNA. Conversely, many lncRNAs display conserved structure between different species while having poorly conserved sequences (Torarinsson et al., 2006). These features of lncRNAs support the hypothesis that they perform biologically robust functions.

**FIGURE 1:**
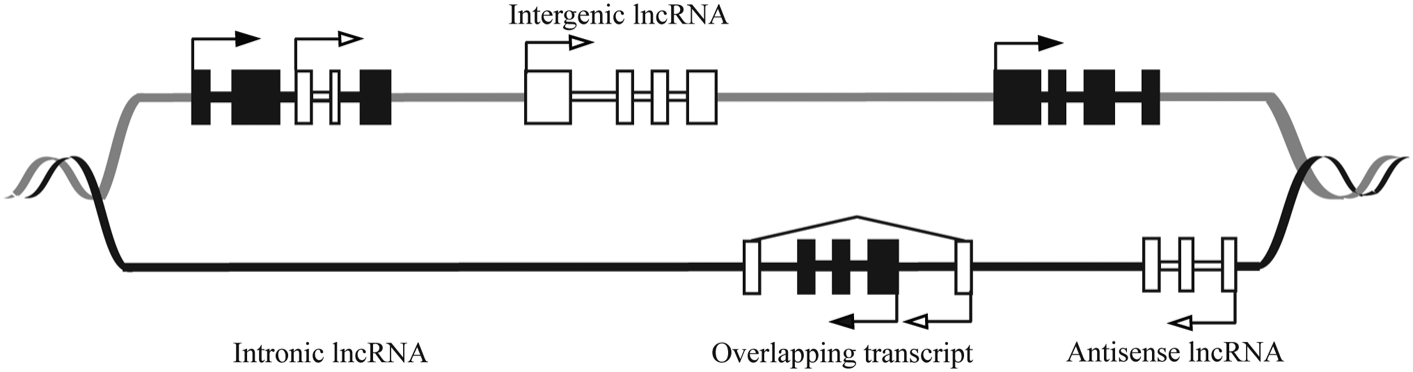
**Schematic showing genomic organization of different lncRNAs.** The DNA strands are shown in black and gray lines. The black and white boxes represent exons of protein coding and lncRNA genes, respectively. LncRNAs are classified as sense (e.g., Intronic or overlapping) or antisense, reflecting the way they overlap with protein coding genes in the same or opposite direction, respectively. The diagram shows that lncRNAs can be antisense to protein coding (antisense lncRNA) or originate from introns of protein coding genes either as by-products of mRNA or as independent transcripts (intronic lncRNA, sense lncRNA). LncRNA also can be transcribed from intergenic regions of the genome (lincRNA). LncRNA genes can overlap the protein-coding genes (overlapping transcript).

Long ncRNAs are further divided into several classes based on their genomic orientation as illustrated in **Figure 1** (Ponting et al., 2009). Despite their abundance, the functions of the majority of lncRNAs have yet to be elucidated. Studies of lncRNA mechanism have revealed extensive variety in their function. LncRNAs can act as transcriptional regulators, acting either in *cis* to regulate nearby genes, or in *trans* to regulate distal genes (Osato et al., 2007; Wang and Chang, 2011; Bak and Mikkelsen, 2014).

**FIGURE 2:**
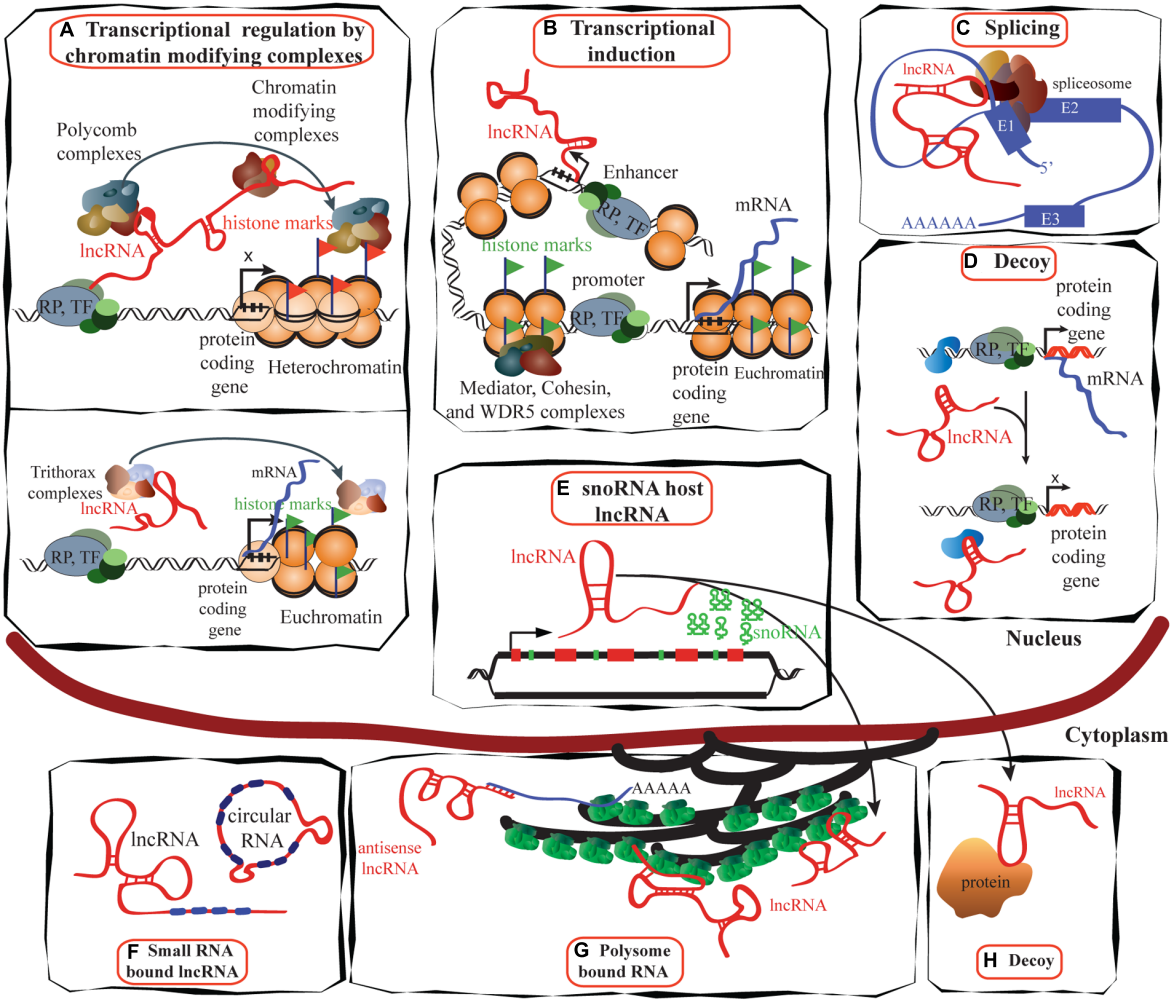
**Schematic illustrating the functions of lncRNAs. (A)** LncRNAs (e.g., *HOTAIR*) can suppress transcription by interacting with PRC1 and PRC2 complexes as well as with other chromatin modifying proteins, maintaining heterochromatin status and suppressing transcription (reviewed in Gibb et al., 2011; upper panel). Trithorax complexes also can interact with lncRNA (e.g., *HOTTIP*) and induce transcription (lower panel; reviewed in Jobe et al., 2012). **(B)** LncRNAs are proposed to be transcribed at enhancer regions and can function in the establishment and maintenance of enhancer–promoter looping and activation of gene expression (Orom and Shiekhattar, 2013). **(C)** LncRNAs such as *MALAT1* can regulate alternative splicing by interacting with the spliceosomal machinery (Tripathi et al., 2013). **(D)** Specific lncRNAs are transcribed and bind to and titrate away protein factors. Decoy lncRNAs can bind to protein factors such as transcription factors and chromatin modifiers. This leads to broad changes in transcriptomes. **(E)** Intronic regions of many lncRNAs host snoRNAs. The snoRNAs derived from these lncRNAs remain in the nucleus while the spliced transcript can move to the cytoplasm and bind either to polysomes or to other proteins (Smith and Steitz, 1998). **(F)** LncRNAs can bind to miRNAs to sequester them and inactivate their repressive functions (reviewed in Gibb et al., 2011). **(G)** Many lncRNAs are associated with polysomes (van Heesch et al., 2014), while the mechanisms of many are yet to be identified, antisense lncRNAs such as *UCHL1AS* regulate the translation of associated mRNAs. **(H)** Decoy lncRNAs such as *GAS5* are present in both in the cytoplasm and the nucleus: *GAS5* translocates from the cytoplasm into the nucleus with glucocorticoid receptor in response to dexamethasone (Kino et al., 2010). TF, transcription factor; RP, RNA polymerase; E1, exon1.

The roles of lncRNA in transcription regulation, mRNA processing, and mRNA maturation are depicted in **Figures 2A–D**. LncRNAs have a broad role in epigenetic control that goes beyond gene regulation at the transcriptional level. Most lncRNA species were previously thought to be restricted to the nucleus, but recent studies have found that a larger proportion than expected is located in the cytoplasm (van Heesch et al., 2014), suggesting post-transcriptional gene regulation, protein shuttling, and localization of proteins and transcripts (**Figures 2E–G**). A large number of lncRNAs associate with actively translating ribosomes (Guttman et al., 2013), indicating that lncRNA regulation of translation may be a common phenomenon, although the exact mechanisms are yet to be understood.

Although only a few lncRNAs have been studied in detail, their characteristic spatial and temporal expression patterns indicate that they play significant but diverse roles in development (Guttman et al., 2011; Ng et al., 2012; Pauli et al., 2012). Furthermore, numerous studies have shown altered expression of lncRNAs in cancer cells compared to normal tissues (Perez et al., 2007; Du et al., 2013; Qiu et al., 2013), indicating that deregulation of these lncRNAs can facilitate disease progression. This review will explore, in particular, the role of lncRNAs in breast development and cancer.

## LncRNAs AS KEY REGULATORY MOLECULES IN DEVELOPMENT AND CANCER

Development of an organism is a complex process, requiring finely regulated gene expression to determine the precise function and architecture of differentiated tissues. Widespread transcription of the human genome and the emergence of lncRNAs as regulatory molecules suggest that development is regulated not only by proteins but also by an expanded network coordinated by RNAs (reviewed in Mattick, 2007). Microarray analyses of embryonic stem cells have revealed many differentially expressed lncRNAs during induced differentiation, confirming their key role in embryonic stem cell development (Dinger et al., 2008). Several of these lncRNAs are transcribed from regions near developmental genes, indicating a complex relationship between protein coding genes and non-protein coding genes in fine tuning development (Dinger et al., 2008). Systematic knockdown of lncRNAs in mouse embryonic stem cells has been shown to have major effects on gene expression patterns, leading to loss of pluripotency and loss of repression of lineage commitment programs. Additionally, the majority of lncRNAs examined interact with transcription factors associated with pluripotency, and ∼30% interact with chromatin (Guttman et al., 2011). The expression of pseudogenes of embryonic transcription factors could facilitate cancer progression. For example, *Oct4* has four related pseudogenes that are found to be expressed in glioma and breast cancer, while *Oct4* is not expressed (Zhao et al., 2011). Such interactions suggest that lncRNAs act as components of networks that include established pluripotency factors and DNA to maintain the undifferentiated replicative state of embryonic stem cells. Chromosome architecture and epigenetic memory are regulated by lncRNAs, which recruit histone modifying complexes and DNA methyltransferases to certain loci, maintaining correct gene expression (reviewed in Mattick, 2007; Amaral et al., 2008).

Alterations of lncRNAs involved in cell homeostatic processes such as cell cycle, DNA damage response, survival, proliferation, and migration have been identified in carcinogenesis. Multiple lncRNAs regulate or are regulated by key cancer pathways, such as the p53 pathway, a master regulator of cell cycle and survival. These include *lincRNA p21* and *PANDA*, which are upregulated upon DNA damage and are induced in a p53-dependent manner (Huarte et al., 2010). *PANDA* interacts with the transcription factor NF-YA to limit expression of proapoptotic genes (Hung et al., 2011). Thus, p53 protein not only induces the activity of lncRNAs to mediate its effects, but is itself regulated by lncRNAs. *MEG3* regulates the activity of p53 by facilitating p53 binding and activation of p53 target gene promoters and inhibiting cellular proliferation (Zhou et al., 2007). LncRNAs affect every hallmark of cancer progression (reviewed in Gutschner and Diederichs, 2012) highlighting the shift from a solely proteomic view of tumor biology to one that includes an extensive network of lncRNAs. Here the roles of lncRNAs involved in mammary gland development and the nature of their deregulation in breast cancer development are discussed.

## ROLE OF lncRNAs IN BREAST DEVELOPMENT AND CANCER

Most organs develop before or around birth, but the mammary gland begins its development in the embryo and continues its growth and differentiation in puberty and adult life (Smith et al., 2012). Mammary gland development in the embryo occurs during mid-gestation and remains quiescent until puberty in which there is further development of the ducts. During pregnancy, the breast tissue undergoes further expansion and branching, and luminal cells rapidly proliferate and differentiate to milk-secreting alveolar cells. These changes underlie the production of milk during lactation. Cessation of lactation is accomplished by involution, in which the breast tissue undergoes extensive apoptosis to return to the quiescent pre-pregnancy state (Watson and Khaled, 2008).

**FIGURE 3:**
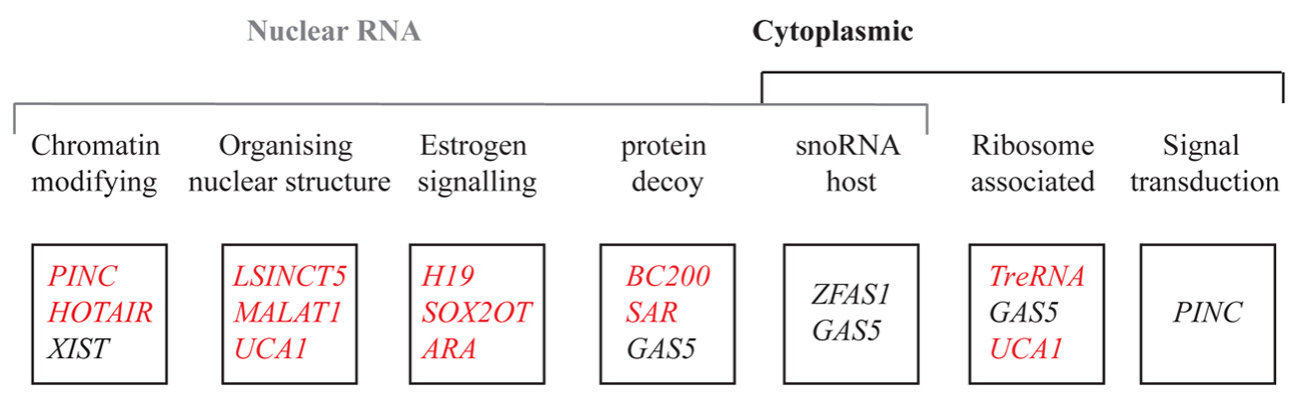
**List of lncRNAs reported in this review based on their localization, function, and expression pattern.** The function of each lncRNA is above each box. The red and black represent the genes upregulated and downregulated respectively in breast cancer.

Breast development is orchestrated by a series of genes that regulate these different cellular processes. Alterations to genes encoding growth factors, nuclear transcription factors, cell cycle regulatory proteins and tumor suppressor proteins have been suggested to create favorable conditions to allow the outgrowth of cells. A major focus in breast cancer research is to understand how normal breast epithelium is transformed into malignant epithelium. With the advent of genome-wide sequencing, it became apparent that it is not solely genetic changes that contribute to breast cancer. Epigenetic changes also play a major part in altering gene expression in such a way as to contribute to cancer (Jones and Baylin, 2007). The role of coding genes in the regulation of breast development is well established (Hennighausen and Robinson, 2001; Anderson et al., 2007; Lim et al., 2010), but less researched is the role of lncRNAs. Large scale microarray analyses using mouse mammary gland RNA from different stages of mammary gland development (Askarian-Amiri et al., 2011), tiling arrays (Perez et al., 2008), and transcriptome profiling (Maruyama et al., 2012) of normal tissues compared to breast cancer tissues, have revealed several lncRNAs which are aberrantly expressed in neoplastic tissues. We describe several lncRNAs that have been implicated in breast cancer development, and describe molecular mechanisms by which they may contribute to normal development and cancer progression (**Figure 3**).

## CHROMATIN REMODELING COMPLEXES

Of those lncRNAs with identified mechanisms, it is apparent that many function as regulators of transcription through chromatin regulation. These lncRNAs act as scaffolds, interacting with target genes and recruiting factors such as histone modifying complexes for gene silencing (reviewed in Rinn and Chang, 2012). The most commonly identified histone modifying complexes that lncRNAs interact with are polycomb group proteins. PcG proteins form multisubunit complexes; the best characterized are polycomb repressor complex 1 (PRC1) and polycomb repressor complex 2 (PRC2), which modify histones by inducing repressive chromatin marks. PRC1 catalyzes ubiquitination of lysine 119 on histone 2A (H2AUb1) while PRC2 methylates lysine 27 on histone 3 (H3K27me2/3). PRC2 is required for the initial targeting of specific gene silencing, whereas PRC1 maintains the gene silencing initiated by PRC2 (Morey and Helin, 2010). LncRNAs also interact with other chromatin modifying complexes, such as the LSD1–CoREST complex, which mediates the removal of H3K4 mono and dimethylation, a chromatin mark associated with gene activation (Rinn et al., 2007). Other chromatin modifying complexes with which lncRNAs associate include G9a (Nagano et al., 2008; Pandey et al., 2008), a H3K9 methyltransferase, and MLL1 (Bertani et al., 2011; Wang et al., 2011), a histone methyltransferase, which associates with activating trithorax complex 1. Through interacting with chromatin modifying complexes, lncRNAs act as guides to target specific gene regions to modify transcription either in *cis* or *trans* (Wang and Chang, 2011). This may be the predominant method in which lncRNAs exert their function, as global analyses have found that a large number of lncRNAs bind to PRC2 and LSD1 (Khalil et al., 2009).

This mechanism of gene regulation by lncRNAs ensures that appropriate genes are switched on or off. Several lncRNAs involved in cancer, including breast cancer, have been shown to promote tumorigenesis by inappropriately silencing genes through deregulated interactions with histone modifying complexes.

### PREGNANCY INDUCED NON-CODING RNA (*PINC*)

Pregnancy induced non-coding RNA (*PINC*) was identified initially in the rat mammary gland and exhibits conserved synteny in the genomes of other mammals. It is differentially expressed as pregnancy progresses (Ginger et al., 2006), highly expressed in the alveolar cells during pregnancy, but declines during the transition from late pregnancy to early lactation, in which the mammary epithelium terminally differentiates to form milk producing cells, and it is again elevated during involution. *PINC* inhibits differentiation, and when it is expressed at low levels, mammary cells are able to undergo differentiation (Shore et al., 2012).

Mouse PINC (*mPINC*) may exert these effects on the mammary gland by regulating cell cycle progression. In HC11 cells, *mPINC* relocates from the nucleus to the cytoplasm as the cell cycle progresses (Ginger et al., 2006), indicative of a role in nuclear–cytoplasmic signal transduction or shuttling. *PINC* interacts with retinoblastoma associated protein 46 (RbAp46), a component of PRC2, suggesting that *PINC* may exert its role in differentiation through modification of chromatin and that it acts to repress gene expression. Several potential target genes, known to be involved in mammary gland development, have been identified, and encode members of the Wnt and Notch signaling pathways (Shore et al., 2012). The role of *PINC* in human mammary gland development and breast cancer has yet to be ascertained.

### HOX TRANSCRIPT ANTISENSE RNA (*HOTAIR*)

HOX transcript antisense RNA (*HOTAIR*) is upregulated in a number of different cancers, including breast (Gupta et al., 2010), colorectal (Kogo et al., 2011), hepatocellular (Geng et al., 2011), and gastrointestinal stromal tumors (Niinuma et al., 2012). In breast cancer, *HOTAIR* expression is upregulated in both primary and metastatic tumors, and its expression in primary tumors is strongly correlated with later metastases, patient prognosis, and death (Gupta et al., 2010).

*HOTAIR* is transcribed in an antisense orientation from the *HOXC* locus. It acts in *trans* as a repressor of the *HOXD* locus by recruiting PRC2, leading to trimethylation of H3K27 and subsequent transcriptional silencing. The bordering regions of *HOXD* are bound by CoREST/REST repressor complexes, which contain the H3K4me2-specific LSD1 demethylase and also maintain appropriate silencing of *HOXD*. *HOTAIR* binds to LSD1 at its 3′ domain, whereas the 5′ domain binds to EZH2 or SUZ12, components of the PRC2. It acts bifunctionally as a scaffold that bridges these two repressor complexes. This results in coordinate binding to their target genes for coupled chromatin remodeling (Tsai et al., 2010). Upregulation of *HOTAIR* leads to genome-wide reprogramming of the PRC2 occupancy pattern from that of breast epithelial cells to that resembling embryonic and neonatal fibroblasts, as well as altered gene expression, that includes the silencing of tumor suppressor genes *JAM2* and *PCDH* (Gupta et al., 2010).

The promoter of *HOTAIR* contains multiple functional estrogen response elements near the transcription start site, and is transcriptionally induced by estrogen, while repressed by tamoxifen. A number of estrogen receptor coregulators also bind to the *HOTAIR* promoter region upon treatment with estrogen such as CBP/p300 and histone methyltransferases MLL1 and MLL3. In the basal state, *HOTAIR* is repressed, but estradiol treatment causes exchange of repressive molecules for transcriptional activators and coregulators, leading to gene activation (Bhan et al., 2013).

An alternative mechanism for *HOTAIR’s* function in breast cancer is through competitive binding with tumor suppressor genes. *BRCA1* is an important breast cancer tumor suppressor gene that functions in DNA damage responses. BRCA1 binds to EZH2, a component of the PRC2 in mouse and human mammary epithelial cells, acting as a competitive inhibitor of *HOTAIR*. Reduced BRCA1 expression, which is often observed in cancer allows *HOTAIR* to bind to PRC2, promoting reprogramming of breast epithelial cells to aggressive cancer cells (Wang et al., 2013).

### XIST

One of the best studied lncRNAs is *XIST*, transcribed from the X inactivation center of the X chromosome that is destined to be inactivated in female mammals (Brown et al., 1991), from whence it spreads along the X chromosome to promote heterochromatin formation and ultimately formation of the Barr body. This mechanism ensures dosage compensation with males. Loss of the Barr body is observed consistently in female cancers including breast cancer. Under normal conditions, two active X chromosomes are incompatible with human life, but activation of both X chromosomes may be advantageous to the cancer cell, as the X chromosome contains several oncogenes. *XIST* expression is lost in female cancers including breast, ovarian, and cervical cancer (reviewed in Froberg et al., 2013). *XIST* itself is under regulation by several lncRNAs such as *Tsix* (negatively; Leeb et al., 2009) and *Jpx* (positively; Tian et al., 2010), highlighting the complexity of the network by which lncRNAs function to induce X chromosome inactivation.

The mechanism by which *XIST* functions in breast cancer is controversial, with some studies showing interaction of the tumor suppressor gene *BRCA1* with *XIST*, and that loss of *BRCA1* is associated with loss of *XIST*. Upon reactivation of *BRCA1*, *XIST* staining by *in situ* hybridization was restored (Ganesan et al., 2002, 2004). Other studies have shown that *XIST* and BRCA1 do not physically co-localize and that reintroduction of BRCA1 did not reactivate *XIST* staining. The same discrepancy of *XIST* and *BRCA1* is observed in human breast tumors, with some studies showing concurrent loss of both *BRCA1* and *XIST* in highly aggressive breast cancers (Richardson et al., 2006) while other studies of breast cancer tissue from patients with germline *BRCA1* mutations show expression of *XIST* in a significant number of samples (Vincent-Salomon et al., 2007). Whether *XIST* interacts with *BRCA1* to regulate cancer progression or if genetic instability due to loss of *BRCA1* expression is responsible for reduced *XIST* expression is yet to be clarified.

## PROTEIN DECOYS

The ability of lncRNAs to form complex secondary structures gives them the capacity to present a diversity of interfaces for binding to proteins. LncRNAs may recruit or organize proteins as evidenced by their roles as chromatin modifiers, or act as decoys which inhibit protein functions. One such lncRNA, *GAS5* which is discussed below, functions as a protein decoy by binding to the glucocorticoid receptor (GR) and preventing it from interacting with the glucocorticoid response element (GRE; Kino et al., 2010; **Figure 2H**).

### BC200

*BC200* is normally selectively expressed in brain tissue and germ cells. It exhibits a high degree of tissue specificity in the brain, showing expression in somatodendritic domains in a subset of neurons but not in other somatic cells. *BC200* functions to repress translation through its oligo(A) rich region which is able to bind polyA binding protein (PABP), so that it is no longer able to bind translation initiation factors (Kondrashov et al., 2005).

This neuronal specificity is lost in a number of tumors, including breast cancer (Chen et al., 1997). *BC200* expression is highly elevated in invasive tumors but not in benign tumors. Furthermore, high *BC200* expression is associated with high nuclear grade, an indicator of aggressive cancer cell behavior. Not all DCIS lesions progress to invasive disease, and *BC200* could be used as a prognostic indicator to assess the invasive potential in early breast tumors to ensure appropriate treatment is given (Iacoangeli et al., 2004).

### STEROID RECEPTOR RNA ACTIVATOR 1 (*SRA1*)

Steroid receptor RNA activator 1 (*SRA1*) acts as a nuclear coactivator for a number of steroid hormone receptors, including the estrogen, progesterone (Lanz et al., 1999), and androgen receptors (Kurisu et al., 2006). It also coregulates the activity of a number of non-steroid receptors and transcription factors. It was discovered as a functional lncRNA which can be alternatively spliced to form a protein coding gene, called steroid receptor RNA activator protein (*SRAP*) which has unique functions as well as overlapping functions to *SRA1* in modulating gene expression. *SRA1* forms a stem-loop structure, which enables it to bind to multiple proteins including steroid receptors, transcription factors, RNA helicases, and other molecules involved in adipogenesis, myogenesis, and adrenal gland gene function (reviewed in Colley and Leedman, 2011).

A mouse model was generated to investigate the relationship between *SRA1* and mammary gland development. No change was observed during early development but in mature virgin transgenic mice, activation of *SRA1* led to an increase in proliferation and differentiation, and to abnormally early development of the ductal epithelium. *SRA1* also regulates cell death, as in these transgenic mice, epithelial hyperplasia was accompanied by increased apoptosis (Lanz et al., 2003). These results underline a relationship between the roles of *SRA1* and of steroid hormone receptors in mammary gland development, as well as a role in regulating apoptosis to maintain healthy breast function. *SRA1* expression is elevated in a number of estrogen responsive tumors such as ovarian (Hussein-Fikret and Fuller, 2005) and breast cancer (Leygue et al., 1999; Murphy et al., 2000). *SRA1* is able to activate the estrogen receptor to increase proliferation and its expression could be indicative of tumor grade or type, as higher expression of *SRA1* was observed in ER-α-positive/PR-negative tumors compared to ER-α-positive/PR-positive tumors (Leygue et al., 1999), but although *SRA1* enhances mammary gland proliferation, it is insufficient for malignant transformation (Lanz et al., 2003). Although the literature suggests that *SRA1* promotes cancer progression, a complex interplay with steroid hormone receptor activation and other oncogenic proteins is likely to be required to drive malignant growth.

## MicroRNA–lncRNA INTERACTIONS

MicroRNAs are small regulatory RNAs of about 22 nt that regulate gene expression by binding to specific mRNAs through sequence complementarity, and targeting them for degradation by the RNA-induced silencing complex (RISC). MiRNAs are regulated transcriptionally and also post-transcriptionally by a class of lncRNAs known as competitive endogenous RNAs (ceRNAs) that act as sponges or decoys to titrate miRNAs away from their target mRNAs and buffer their activity (Bak and Mikkelsen, 2014). Several ceRNAs such *HULC* in liver cancer cells sequester miR-372 (Wang et al., 2010) and *PTENP1* sequesters *PTEN*-targeting miRNAs through similarities in miRNA binding sites in prostate cancer cells (Poliseno et al., 2010). Non-protein coding regions of the genome contain a high proportion of miRNA targets, and as lncRNAs are often found in intergenic and intronic regions of the genome, could be frequently targeted by miRNAs (Clark et al., 2014). Alignment of several other genes and their corresponding ceRNAs shows that miRNA binding sites are well conserved indicating that miRNA–lncRNA interactions play major roles in biological processes (Poliseno et al., 2010). MiRNAs can target the exons of coding regions and exon–exon junctions, enabling selective targeting of particular splice variants (Tay et al., 2008).

Long ncRNAs, ceRNAs, and miRNAs form regulatory networks across the transcriptome, communicating with each other to regulate gene expression. This includes short motifs known as pyknons, which are a subset of variable length patterns. Pyknons are conserved in the genome, and are found in coding and non-coding regions, but mostly in 3′UTRs. Pyknon sequences contain 40% of known miRNA sequences, suggesting that they regulate miRNA function (Rigoutsos et al., 2006). Different sets of pyknons are associated with disease-associated single nucleotide polymorphisms, suggesting that susceptibility to disease is due to genome wide regulation of miRNAs (Glinsky, 2009).

While some established miRNA–lncRNA interaction sites are conserved to various extents, others lack conservation despite showing confirmed functions (Alaei-Mahabadi and Larsson, 2013). It may potentially be difficult to extrapolate gene effects in animal models to human diseases. To address this challenge, it must first be demonstrated that miRNA binding sites in lncRNAs are conserved before assuming cross-species efficacy (Jackson and Levin, 2012). Paci et al. (2014) have used computational approaches to assess whether specific lncRNAs function as miRNA decoys in normal and invasive breast epithelium. In normal breast tissue, a complex regulatory network of miRNA-mediated interactions exist to bridge target mRNAs and lncRNAs, and this is missing in tumor samples. However, the role of miRNA-decoys in breast cancer remains to be validated with functional studies.

## SnoRNA HOSTS

Small nucleolar RNAs are a well characterized class of small ncRNAs that predominantly localize to the nucleolus and act there as guides for post transcriptional modification of ribosomal RNAs. These snoRNAs bind modifying proteins, then hybridize with specific rRNA sequences, facilitating rRNA modification. Two major classes of snoRNAs are involved in two different types of post-transcriptional modification: the C/D box snoRNAs which define sites for 2′O ribose methylation, and the H/ACA box snoRNAs which define the sites for pseudo-uridylation (Williams and Farzaneh, 2012).

Genetic searches for cancer genes have revealed that aside from their housekeeping function for rRNA modifications, a number of snoRNAs are also deregulated in cancer. An example is the snoRNA U50, a candidate tumor suppressor gene. It is downregulated and frequently mutated in prostate and breast cancer (Nallar and Kalvakolanu, 2013).

Small nucleolar RNAs are often located within the introns of protein coding and lncRNA genes. The latter may have independent functions, such as involvement in protein synthesis, and may contribute to breast cancer progression.

### ZNFX1-ANTISENSE RNA 1 (*ZFAS1*)

ZNFX1 antisense RNA 1 *(ZFAS1)* is an antisense lncRNA that overlaps the promoter region of the gene *ZNFX1*, which encodes a zinc finger protein whose exact role remains to be elucidated. *ZFAS1* is host to three snoRNAs in three consecutive introns. *ZFAS1* is expressed in a wide variety of tissues, showing highest expression in the mammary gland and lung. It contributes to mammary gland development, showing enrichment in the alveolar structures and ducts and differential expression during different stages of pregnancy in mouse mammary glands. As well as acting as a snoRNA host, *Zfas1* has independent regulatory functions. *Zfas1* is expressed in both the nucleus and cytoplasm, and knockdown of cytoplasmic *Zfas1* by siRNA in mouse mammary epithelial cell lines increases proliferation. This suggests that *Zfas1* has dual functions: after splicing in the nucleus to generate snoRNAs, *Zfas1* is transported to the cytoplasm where it regulates cellular proliferation. In addition to regulating mammary gland development, *ZFAS1* contributes to breast cancer, as its expression is reduced in human ductal carcinoma compared to normal breast epithelium. *ZFAS1* plays a role in proliferation as well as differentiation, and its down regulation may promote cancer progression, indicating that it may have tumor suppressor functions (Askarian-Amiri et al., 2011).

### GROWTH ARREST-SPECIFIC 5 (*GAS5*)

Long ncRNA growth arrest-specific 5 (*GAS5*) containing up to 12 exons was first isolated from growth arrested NIH-3T3 cells, induced upon serum starvation of cells (Schneider et al., 1988; Raho et al., 2000). *GAS5* contains a stem-loop structure at its 3′ end that resembles the double stranded GRE and is able to act as a decoy for the GR, binding to it and sequestering it from its DNA response element. *GAS5* suppresses the transcription of GR target genes, such as *cIAP2*, *PEPCK*, and *G6Pase*. These genes are crucial for glucose metabolism, and thus *GAS5* may function to conserve energy resources in response to starvation (Kino et al., 2010).

Growth arrest-specific 5 acts as a tumor suppressor gene in breast cancer as its expression is significantly reduced compared to normal breast epithelium. It is a regulator of apoptosis as forced overexpression in breast cancer lines induced apoptosis, sensitized cells to apoptotic inducers and repressed proliferation (Mourtada-Maarabouni et al., 2008). *GAS5*’s function as a tumor suppressor gene is a combination of its two mechanisms of action. Decreased expression of *RNU44*, a snoRNA derived from *GAS5* is associated with more aggressive tumors in breast cancer and head and neck squamous cell carcinoma (Gee et al., 2011). Additionally, many GR target genes are anti-apoptotic (Kino et al., 2010) and through its inhibition, *GAS5* promotes apoptosis.

## ORGANIZING NUCLEAR STRUCTURES

The nucleus is highly organized into different domains, which provide an essential basis for the complex regulation of nuclear processes and gene expression. In addition to protein factors, some of these subnuclear domains contain lncRNAs. These nuclear lncRNAs are essential for nuclear regulation, with some acting as structural scaffolds, while others directly modulate nuclear compartment function. Nuclear organization is fundamental to modulating the activities of the genome, and this can become disrupted in cancer (Bergmann and Spector, 2014). In breast cancer, lncRNAs involved in organizing nuclear structures become deregulated, and may drive cancer progression, as discussed below.

### LONG STRESS INDUCED NON-CODING TRANSCRIPT 5 (*LSINCT5*)

Long stress induced non-coding transcript 5 (*LSINCT5*) is a nuclear localized lncRNA that was discovered in screens to identify lncRNAs upregulated upon treatment with stress inducing chemicals. In normal tissues, *LSINCT5* is highly expressed in proliferative and stress responsive tissues such as the colon and spleen compared to non-proliferative tissues such as brain. Expression of *LSINCT5* is increased in several cancers, including breast and ovarian cancer. Because of its high expression in proliferative tissues and cancer, *LSINCT5* could potentially function as a regulator of proliferation. This is supported by *LSINCT5* knockdown in breast and ovarian cancer cell lines, which impairs proliferation (Silva et al., 2011). Additionally, knockdown of *LSINCT5* leads to altered expression of several genes including suppression of *CXCR4,* a breast cancer marker associated with metastasis, the lncRNA *NEAT1* and the protein coding gene *PSPC1* (paraspeckle component 1; Silva et al., 2011). *LSINCT* co-localizes with *NEAT1* in the nucleus (Smith and Jessica, 2012), suggesting *LSINCT5* regulates nuclear paraspeckle maintenance. How *LSINCT5* contributes to paraspeckle function is yet to be determined, and it is possible that it acts to regulate proliferative genes in specific nuclear bodies.

### METASTASIS ASSOCIATED LUNG ADENOCARCINOMA TRANSCRIPT 1 (*MALAT1*)

Metastasis associated lung adenocarcinoma transcript 1 (*MALAT1*) is a highly conserved, abundant lncRNA whose expression is localized to the nucleus. It was discovered in non-small cell lung cancer as a prognostic marker of later metastasis (Ji et al., 2003) but since then has been found in several other types of human cancer, including breast cancer. *MALAT1* expression is typically upregulated in cancer and has been associated with several neoplastic phenotypic features including proliferation, cell death, migration, invasion, and metastasis (reviewed in Gutschner et al., 2013b).

Metastasis associated lung adenocarcinoma transcript 1 is a core component of nuclear speckles, which are involved in mRNA processing, splicing, and export (Hutchinson et al., 2007). Blocking transcription causes a redistribution of *MALAT1* from the nuclear speckles to the nucleoplasm (Bernard et al., 2010), indicating that *MALAT1* regulates active transcription or processing. *MALAT1* also localizes with several splicing factors, including SRSF1–3 (Anko et al., 2012). *MALAT1* depletion decreases the association of these splicing factors with the nuclear speckles and differential alternative splicing of pre-mRNA (Tripathi et al., 2013). It is proposed that *MALAT1* regulates the recruitment of splicing factors to these pre-mRNAs. *MALAT1* also regulates other nuclear factors, such as polycomb group proteins. This lncRNA binds to unmethylated Polycomb 2 protein (PC2) in response to growth signals, shifting its location from repressive polycomb bodies on growth control gene loci to active interchromatin granules, activating their expression. *MALAT1* acts as a switch for polycomb target genes from repressive nuclear domains to active nuclear domains, influencing gene expression (Gutschner et al., 2013b).

Metastasis associated lung adenocarcinoma transcript 1 also regulates expression of cell cycle genes. It is required for G1/S transition and mitotic progression, and depletion of *MALAT1* leads to cell cycle arrest and activates p53 and p53 target genes. Additionally, cells depleted in *MALAT1* show reduced expression of *B-MYB*, an oncogenic transcription factor involved in G2/M progression. *MALAT1* regulates the cell cycle by modulating key transcription factors which, when deregulated, could promote cancer progression (Tripathi et al., 2013).

In breast cancer, *MALAT1* is also upregulated (Guffanti et al., 2009), although its function remains to be determined. *MALAT1* harbors mutations and deletions in luminal breast cancer, in a region which modulates interaction with the splicing factors 1–3 (SRSF1–3; Gutschner et al., 2013b). These mutations could alter the function *of MALAT1*, leading to deregulated splicing events. Treatment of breast cancer cells with high levels of estradiol reduces *MALAT1* transcription, reducing proliferation, migration, and invasion (Zhao et al., 2014). Although higher expression of *MALAT1* is associated with poorer outcomes in a number of cancers, Sun et al. (2009) have shown that *MALAT1* is decreased in proliferating mammary glands of c-myc transgenic mice and further decreased to undetectable levels in mammary tumors. *MALAT1* may regulate many genes, and its regulation is context specific.

## RIBOSOME ASSOCIATED lncRNAs

The advent of high throughput technologies is uncovering unexpected roles for lncRNAs. Ingolia et al. (2011) performed genome wide ribosome profiling in mouse embryonic stem cells, a recently developed technique that involves digesting RNA and sequencing the portion bound to the 80S ribosome to give a profile of ribosome occupancy. Unexpectedly, they discovered that a large set of intergenic lncRNAs was bound to ribosomes, raising the question of whether these lncRNAs were translated to small proteins. In light of these findings, Guttman et al. (2013) revisited these experiments with a new metric to discriminate between transcripts that were being actively translated and those just bound to the ribosome. During translation, ribosomes release their transcript upon encountering a stop codon, which is seen as a drop in 3′UTR occupancy. As this should not occur for non-coding transcripts, Guttman et al. (2013) developed a metric based on ribosome release of these transcripts. What they found was that even though a large number of long intergenic ncRNAs (lincRNA) bind, they are not actively translated. This is not just restricted to lincRNAs, as independent studies have also found a large proportion of other classes of lncRNAs bound to actively translating ribosomes (van Heesch et al., 2014).

As these lncRNAs have been confirmed to not encode for proteins, this raises the question of why so many lncRNAs are bound to ribosomes. Few of these lncRNAs have been studied in detail, but those that have been investigated have been found to regulate translation by associating with polysomes during stress conditions and either upregulating specific proteins, as is the case for *Uchl1AS* (Carrieri et al., 2012), or globally inhibiting translation as for *mlonRNAs* found in yeast (Galipon et al., 2013). Their roles in disease processes have not been established. Here, we highlight ribosome associated lncRNAs and discuss their putative roles in regulating ribosome function in breast cancer.

### TRANSLATIONAL REGULATORY lncRNA (*TreRNA*)

Translational regulatory lncRNA (*TreRNA*) was identified through genome wide computational analysis (Orom et al., 2010) and found to be upregulated in cellular invasion and tumor metastasis, as well as upregulated in lymph node metastatic breast cancer samples when compared to matched primary tumors. *TreRNA* is also overexpressed in primary colon cancer samples compared to healthy controls, indicating an association between *treRNA* expression and cancer progression, including metastatic development (Gumireddy et al., 2013).

Further evidence that *treRNA* promotes invasion and metastasis has been obtained from *in vitro* and *in vivo* studies. Enforced expression of *treRNA* in breast cancer cell lines and mouse models significantly promoted invasion and lung metastases, respectively, whereas knockdown suppressed cellular migration, invasion, and metastasis in mouse (Gumireddy et al., 2013). *TreRNA* also leads to suppression of epithelial markers, which is associated with metastasis and transition to a mesenchymal state. Enhanced *treRNA* expression suppresses E-cadherin, and to a lesser extent zonula occludens-1 (ZO-1) and β-catenin, and increases expression of fibronectin and vimentin (Gumireddy et al., 2013). This change in cellular markers indicates a partial epithelial to mesenchymal transition.

This lncRNA employs different mechanisms of action depending on its cellular location. In the nucleus, *treRNA* acts as an enhancer for neighboring genes such as *Snail* (Orom et al., 2010). Cytoplasmic *treRNA* on the other hand acts to regulate E-cadherin by modulating its translation. Expression of *treRNA* reduces the level of E-cadherin protein without affecting mRNA abundance, and redistributes E-cadherin mRNA from high molecular weight polysomes to low molecular weight polysomes, strongly indicating that it suppresses translation of E-cadherin. How *treRNA* functions to inhibit translation is yet to be known, but it is proposed that *treRNA* promotes the formation of a ribonucleoprotein complex with hnRNP K, *FXR1*, *FXR2*, *PUF60,* and *SF3B*. This *treRNA*–protein complex may interact directly or indirectly with E-cadherin mRNA to inhibit its translation (Gumireddy et al., 2013).

## LncRNAs IN ESTROGEN SIGNALING

Estrogens are mitogens that promote cellular growth and proliferation, and development of the mammary gland. Estrogens signal by binding to nuclear receptors that regulate gene transcription. Mice lacking estrogen receptors cannot undergo pubertal mammary gland development. Aberrant estrogen signaling participates in breast cancer. Increased estrogen exposure may stimulate excess proliferation and has been linked to higher rates of breast cancer. 60–70% of breast cancers overexpress the estrogen receptor and show increased responses to the mitogenic effects of estrogen (reviewed in Manavathi et al., 2013). Estrogen directly modifies the expression of protein coding genes and also interacts with certain lncRNAs to modify gene expression, as discussed below.

### H19

*H19* is derived from a large imprinted locus on human chromosome 11p15.5. This lncRNA is abundantly expressed in the developing embryo, highly expressed in the mammary bud and adjacent tissues and differentially regulated during mammary gland development. *H19* is expressed exclusively from the maternal allele where it acts to regulate the expression of *IGF2*, the paternally expressed growth factor from the same locus, by repressing both its transcription (Wilkin et al., 2000) and translation (Li et al., 1998). *H19* is transcriptionally induced by the transcription factor E2F1, which promotes cell cycle progression. Its expression is repressed by pRB and E2F6, both of which are E2F-dependent transcriptional inhibitors (Ratajczak, 2012). c-Myc, which binds to E-box regions, also promotes transcription of *H19* (Barsyte-Lovejoy et al., 2006). As *H19* is under the transcriptional control of two well-known oncoproteins, E2F1 and c-myc, it is likely that it also participates in a network that promotes tumor progression.

*H19* expression is normally downregulated in normal adult tissue, with only a few tissues, including the mammary gland, showing basal expression. In mouse mammary glands *H19* expression is repressed during prepubertal development, upregulated during both puberty and pregnancy, repressed during lactation and then once again upregulated during involution indicating that it is not necessary for terminal differentiation but may instead function in proliferation, migration, and pre-terminal differentiation (Ratajczak, 2012). *H19* is upregulated in breast cancer, with over 70% of breast adenomas showing overexpression, and is significantly correlated with ER+ and PR+ breast cancers (Adriaenssens et al., 1998). Estradiol transcriptionally regulates *H19*, as treatment with estradiol of ovariectomized and adrenalectomized mice increases *H19* expression in the uterus, as well as increasing its expression in breast cancer cell lines (Adriaenssens et al., 1999). Transfection of *H19* in breast cancer cells also increases their ability to form tumors when injected into mice (Lottin et al., 2002) and its overexpression leads to an increase in breast cancer proliferation by increasing entry into S phase, whereas knockdown prevented cell cycle progression through S phase (Berteaux et al., 2005).

### SOX2 OVERLAPPING TRANSCRIPT (*SOX2OT*)

SRY (sex determining region Y)-box 2 (*SOX2*) is a transcription factor that acts a key regulator of pluripotency and lies within the intron of a lncRNA, called *SOX2OT. SOX2* is required for embryonic stem cell self-renewal and pluripotency and is often deregulated in cancer (Liu et al., 2013). *SOX2OT* is a stable transcript, which, like *SOX2*, is expressed in embryonic stem cells and downregulated upon induction of embryoid body differentiation. *SOX2OT* is dynamically regulated during embryogenesis of several vertebrates, and restricted to the brain in adult mouse and humans (Amaral et al., 2009).

In breast cancer, *SOX2* overexpression is observed in aggressive tumors and associated with a stem cell like phenotype and *SOX2OT* plays a key role in regulating *SOX2*. Expression of both *SOX2* and *SOX2OT* are positively correlated in breast cancer samples, with ER+ tumors showing greater expression of both genes than ER- tumors, suggesting that this lncRNA is sensitive to steroid hormones such as estrogens. Ectopic expression of *SOX2OT* induced *SOX2* expression, suggesting a positive regulatory role for *SOX2OT*. Orthotopic xenografts of breast cancer cells also show upregulation of both *SOX2* and *SOX2OT* (Askarian-Amiri et al., 2014). *SOX2OT* not only regulates *SOX2* in vertebrate development, but also regulates *SOX2* during disease processes such as cancer.

## LncRNAs AND DRUG RESISTANCE

Survival rates of patients with breast cancer have significantly improved over the past several decades. While many factors are credited for this more positive outlook, including early detection methods, this improvement can also be attributed to new treatment programs as well as the development of new drugs. However, continuous chemotherapy often leads to drug resistance, hindering the long-term control of cancers. Chemoresistance is often reported to be a result of increase in eﬄux transporters or deregulation of signaling pathways, and there is increasing evidence that epigenetic factors contribute to drug resistance. In the following section lncRNAs that contribute to drug resistance in breast cancer will be discussed.

### ADRIAMYCIN RESISTANCE ASSOCIATED (*ARA*)

Doxorubicin, also known as Adriamycin, is a chemotherapeutic drug that acts as a topoisomerase II poison. It is used to treat a variety of cancers, including breast cancer (Cortes-Funes and Coronado, 2007). However, prolonged exposure to doxorubicin induces drug resistance, making the cancer harder to treat. Jiang et al. (2014) recently identified several lncRNAs including *ARA* that showed differential expression in doxorubicin resistant breast and liver cancer cell lines. This lncRNA is derived from the intronic region of the *PAK3* gene, and is upregulated in doxorubicin resistant MCF7 cells compared to the parental cell line. Treatment of sensitive breast cancer cell lines with doxorubicin led to an increase in *ARA* expression, and thus prolonged exposure to doxorubicin may lead to constant upregulation of *ARA* during the initiation of drug resistance. *ARA* may also be involved in self-sufficiency in estrogen signaling, as estrogen receptor negative breast cancer cell lines expressed greater levels of *ARA* than estrogen receptor positive cell lines.

Silencing of *ARA* in doxorubicin resistant breast cancer cell lines restored sensitivity to doxorubicin, as well as inhibiting cellular proliferation, inducing apoptosis and the accumulation of cells in the G2/M phase and reducing cell motility. Genes regulated by *ARA* are enriched in several pathways, including MAPK, focal adhesion, PPAR, and metabolism signaling pathways. *ARA* may contribute a survival and proliferative advantage to doxorubicin resistant cells, and inhibition of *ARA* returns the breast cancer cell to the parental drug sensitive phenotype (Jiang et al., 2014).

### UROTHELIAL CARCINOMA-ASSOCIATED 1 (*UCA1*)

Urothelial carcinoma-associated 1 (*UCA1*) was originally identified as being dramatically upregulated in bladder cancer, and is known as a highly specific and sensitive biomarker for diagnosing bladder carcinoma (Wang et al., 2008). *UCA1* expression is elevated in embryonic tissues, then turned off in most tissues after birth. It is reactivated in cancer, indicating that *UCA1* is an oncofetal gene (Wang et al., 2008). In breast cancer, *UCA1* is upregulated and displays oncogenic properties. *In vitro*, ectopic expression of *UCA1* enhances breast cancer cell growth, while *UCA1*-siRNA suppresses growth, leading to the accumulation of cells in G1 phase, as well as decreased Ki67 staining in tumors from mice (Huang et al., 2014). An isoform of *UCA1* is associated with doxorubicin resistance in a squamous cell carcinoma line (Tsang et al., 2007), but it is not known whether this applies to breast cancer.

Urothelial carcinoma-associated 1 forms a ribonucleoprotein complex with heterogeneous nuclear ribonucleoprotein 1 (hnRNP-1). Its expression is mainly restricted to the nucleus, where it plays a key role in the splicing of pre-mRNA (Izquierdo et al., 2005), but low levels are also expressed in the cytoplasm. In the cytoplasm, hnRNP1 acts as an activator of translation. By binding to the 5′ UTR of mRNA, hnRNP recruits ribosomal proteins to initiate translation of several mRNAs such as that of p27, a well-known tumor suppressor protein that inhibits cyclin-dependent kinases. *UCA1* inhibits the interaction between the p27 mRNA and hnRNP, preventing the translation of p27. Thus, there exists a competition between p27 and *UCA1*, as indicated by a decrease in p27 mRNA and hnRNP complex when *UCA1* is overexpressed. Additionally, although both *UCA1* and p27 mRNA interact with hnRNP1, they are present as separate hnRNP complexes, perhaps indicating that they compete for the same binding site, or that binding of either RNA in an allosteric site induces a conformational change that prevents binding of the other (Huang et al., 2014).

## LncRNAs AS POTENTIAL THERAPEUTIC TARGETS

As discussed in this review, several lncRNAs have been implicated in cancer and modulation of these lncRNAs could lead to new therapeutic advances for cancer treatment.

Long ncRNAs can be targeted by RNA interference technologies such as siRNAs and shRNAs. These molecules show a high degree of selectivity and knockdown efficiency and could therefore be used to silence lncRNAs for treatment of disease. Although the use of siRNA and shRNA for therapeutic use is still in development, a few siRNAs have been used in clinical trials for a number of diseases, including cancer (Burnett et al., 2011). Various chemical modifications can also improve the stability and delivery of these RNA targeting molecules, which would be valuable if they were to be used in a clinical setting to target critical lncRNAs (Lorenz et al., 2004).

Other strategies that could be used to target lncRNAs include antisense oligonucleotides (ASO), single stranded DNA or RNA molecules with a specific sequence designed to target a particular RNA. ASOs bind to their target RNAs with high sequence specificity, and induce their degradation by RNAse H1. They have been used experimentally to inhibit *MALAT1* in cancer cells and subcutaneous tumors in nude mice (Gutschner et al., 2013a) with success. ASOs could be designed to target other lncRNAs which act as oncoRNAs so as to inhibit their expression.

Long ncRNAs have also been proposed as potential biomarkers for cancer progression. As discussed previously, *HOTAIR* and *BC200* are upregulated in breast cancer as well as in other cancers. High expression of *HOTAIR* is correlated with poor survival in breast cancer, increased recurrence of hepatocellular carcinoma following surgical hepatectomy, and high grades of gastrointestinal stromal tumor (GIST; Gupta et al., 2010; Niinuma et al., 2012; Ishibashi et al., 2013). *HOTAIR* and *BC200* (Iacoangeli et al., 2004) may be useful to predict patient outcome and tumor aggressiveness in breast cancer.

However, therapeutic use of lncRNAs is not without its limitations. New tools will be needed to study their function, of which RNA interference technologies will be vital. Nevertheless, identifying the mechanism of lncRNAs will require extensive characterization of individual cases. These studies will also need to address the biological context of lncRNAs, as lncRNAs might regulate different functions in different tissues or disease processes.

Long ncRNAs are expressed at low abundance compared to protein coding genes, which can make functional studies challenging. However, this may be due to their expression in only small subpopulations of cells, providing highly specific targets for disease therapy (Djebali et al., 2012). LncRNAs show conserved function and structure in different species, and despite their poor sequence conservation could potentially be targeted for drug therapy. However, extrapolating results in animal models to humans must be done with care, especially for those lncRNAs whose roles depend on their sequence conservation such as miRNA decoys.

LncRNAs are active in multiple levels of gene regulation. They play important roles in the human mammary gland development and breast carcinogenesis. Several studies have examined their role as therapeutic targets and it is possible that lncRNAs could be targeted clinically in the future.

## CONCLUDING REMARKS

Long ncRNAs are important regulators of gene expression and have a wide variety of functions that enable them to interact with multiple pathways that modulate breast cancer progressions. These lncRNAs have been shown to act as oncoRNAs, often upregulated in breast cancer and facilitating tumor growth and progression, or acting as tumor suppressors, showing downregulation in breast cancer and inhibiting tumorigenesis (**Table 1**). Their role as therapeutic agents is currently being explored, and highly specific lncRNA expression signatures make them attractive markers for accurate disease diagnostics. As well as their use for diagnosis and prognosis, the advancement of RNA-based therapeutics opens up lncRNAs as new targets for therapy. The functional role of the plethora of lncRNAs is only beginning to be elucidated, and provides an untapped resource for the development of cancer therapies and biomarkers.

**Table 1:** List of lncRNAs involved in breast cancer progression with their proposed functions.

LncRNA	Expression in breast cancer	Mechanism of action	Effect on breast cancer progression
*ARA*	Upregulated in doxorubicin resistant breast cancer cells	Unknown, but it may initiate doxorubicin resistance, perhaps through MAPK, PPAR, focal adhesion and metabolism signaling pathways	Silencing of *ARA* increases sensitivity to doxorubicin, inhibits cellular proliferation, induces apoptosis, reduces cell motility
*BC200*	Upregulated	Sequesters PABP, preventing protein synthesis	Promotes aggressive cell behavior, as expressed only in invasive tumors
*GAS5*	Downregulated	•Acts as a decoy for GRE and sequesters GR •Host for several snoRNAs	Resistance to apoptosis
*H19*	Upregulated	Unknown, but may be involved in cell cycle progression as it is induced by E2F1 and c-Myc	•Promotes tumor growth when transfected cells are injected into mice •Increases entry into S phase of cell cycle
*HOTAIR*	Upregulated	•Trimethylation of histone H3K27 by recruiting PRC2 to *HOXD* locus •Demethylation of H3K4 by binding to LSD1 at *HOXD* locus •Competitive inhibitor of BRCA1 binding to EZH2 •Silences *PTEN* through methylation	•Increased invasion *in vitro* Increased metastasis *in vivo* •Predictor of poor prognosis
*LSINCT5*	Upregulated	Localizes with *NEAT1* and regulates nuclear paraspeckles	Increases cellular proliferation in response to stress
*MALAT1*	Upregulated	•Regulates active transcription •Recruits splicing factors •Regulates expression of cell cycle genes •Modulates cellular location of PC2	Unknown, but in other cancers, overexpression increases cell motility and migration, and acts as a predictor for metastasis
*SOX2OT*	Upregulated	Induces *SOX2* expression	Unknown, but may promote EMT through induction of *SOX2*
*SRA1*	Upregulated	Binds to corepressors and coactivators to modulate activity of estrogen receptor	Increases cellular proliferation by activating estrogen receptor
*TreRNA*	Upregulated	•Nuclear treRNA acts an enhancer for Snail transcription •Cytoplasmic treRNA inhibits the translation of E-cadherin, possibly through formation of a ribonucleoprotein complex with hnRNP K, FXR1, FXR2, PUF60 and SF3B	Promotes invasion and metastasis, through regulation of Snail and E-cadherin, may promote epithelial-mesenchymal transition
*UCA1*	Upregulated	Competes with p27 for binding to hnRNP-1, preventing p27 translation	Increases breast cancer growth by promoting cell cycle progression
*XIST*	Downregulated	•X chromosome inactivation to form Barr body •May interact with BRCA1 but mechanism unknown	Loss of *XIST* could be a result of genetic instability, tumors that lose *XIST* are highly aggressive

## Conflict of Interest Statement

The authors declare that the research was conducted in the absence of any commercial or financial relationships that could be construed as a potential conflict of interest.
